# Complete Genome Sequence of Mycobacteriophage Joy99

**DOI:** 10.1128/MRA.00556-21

**Published:** 2021-08-12

**Authors:** Faith Cox, Josh Katuri, Megan Adams, Danielle Bachhofer, Ashleigh Cooper, Jessica Doty, Miranda Fuentes, Leeila Hanson, Travis Miller, Jonathan Musgrave, Aleksey Palumbo, Camille Trautman, Julie Edwards, Dustin Edwards

**Affiliations:** a Department of Biological Sciences, Tarleton State University, Stephenville, Texas, USA; Portland State University

## Abstract

Joy99 is a siphoviral mycobacteriophage with a 59,837-base pair double-stranded DNA genome and is predicted to contain 97 protein-coding genes and a single tRNA gene. Joy99 was isolated in Saint Louis, MO, and annotated by students at Bluff Dale High School in community engagement with Tarleton State University.

## ANNOUNCEMENT

In 2019, 10 million Mycobacterium tuberculosis infections occurred worldwide (1). An estimated half million infections were rifampin-resistant tuberculosis, and 206,030 were multidrug-resistant tuberculosis (MDR-TB), a 10% increase from 2018 (1). Characterized bacteriophages could offer an alternative treatment option for MDR-TB (2, 3). Mycobacteriophage Joy99 was discovered in soil from a municipal mulch pile in Saint Louis, MO (global positioning system [GPS] coordinates 38.664567 N, 90.3179 W) (4, 5). Soil samples were incubated in 7H9 liquid medium at 37°C for 2 h before the supernatant was centrifuged, filtered through a 0.22-μm filter, and incubated with Mycobacterium smegmatis mc^2^ 155 at 37°C on Luria agar plates. Joy99 was isolated by two rounds of picking a single, well-separated plaque, followed by diluting the bacteriophage sample in a 10-fold dilution series and plating it with M. smegmatis mc^2^ 155 (6).

**FIG 1:**
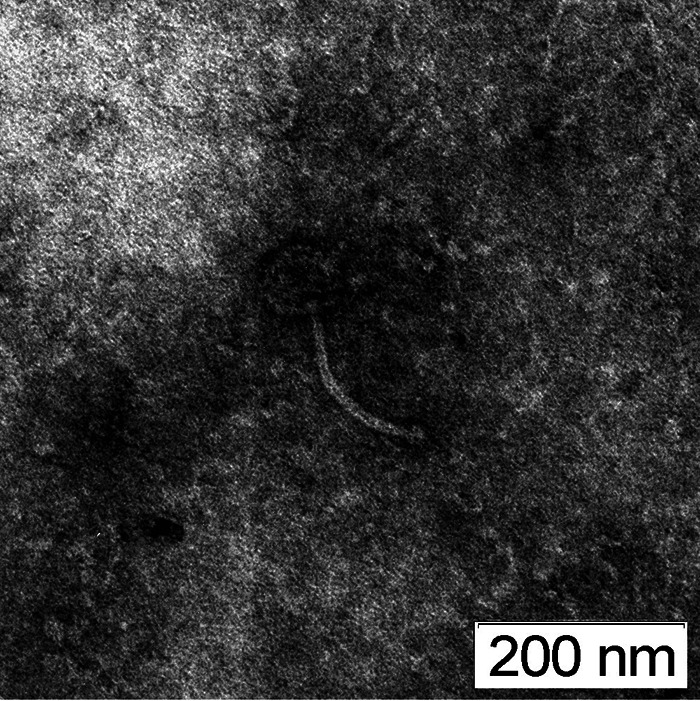
Transmission electron microscopy (TEM) of mycobacteriophage Joy99. High-titer lysate on a carbon type B 300-mesh grid was negatively stained with 1% uranyl acetate. A TEM micrograph of mycobacteriophage Joy99 shows an approximately 75-nm-diameter nonenveloped icosahedral capsid and a 180-nm flexible noncontractile tail.

Joy99 plaques formed a three-ring morphology with a clear center spot, thin middle ring, and turbid outer ring. Negative-staining transmission electron microscopy with 1% uranyl acetate on a carbon type B 300-mesh grid showed *Siphoviridae* morphology (4). ImageJ v1.53h (7) measured a 75-nm-wide isometric capsid and 180-nm-long tail (Fig. 1). High-titer lysate was obtained by flooding “webbed” plates, as described in the Phage Discovery Guide, and DNA extraction was performed at Washington University using the Promega Wizard DNA clean-up system (8). Sequencing libraries were prepared from genomic DNA using a 454 DNA library prep kit and sequenced with a Roche 454 GS FLX instrument at the Pittsburgh Bacteriophage Institute to approximately 157-fold coverage from 17,587 total single-end reads with an average read length of 165 bases (9). The single-bacteriophage contig assembly was performed using Newbler v2.7, and the assembly was checked for completeness, accuracy, and genome termini using Consed v22.0 software (9, 10). Joy99 has a linear double-stranded DNA genome that is 59,837 bp long with 66.6% G+C content and an 11-base 3′ sticky overhang composed of 5′-CTCGTAGGCAT-3′.

Whole-genome nucleotide alignment using BLASTn (https://blast.ncbi.nlm.nih.gov/) indicated that Joy99 has nucleotide sequence identity of more than 99% with other subcluster K1 bacteriophages (Table 1) (11). Initial auto-annotations were generated using Glimmer v3.02 (12) and GeneMark v2.5p (13) and were revised manually using DNA Master v5.23.2 (http://phagesdb.org/DNAMaster/) and PECAAN (https://pecaan.kbrinsgd.org/). Gene functions were assigned using HHpred v3.0beta (14, 15) and BLASTp (11). The tRNA was characterized using ARAGORN v1.2.38 (16) and tRNAscan-SE v2.0 (17). All tools were run with default parameters. The Joy99 genome contains 97 predicted protein-coding genes and a single tRNA gene for tryptophan; 14.3% of K1 bacteriophages encode a tRNA. The start codon usage is 58.7% AUG, 40.2% GUG, and 0.1% UUG. Like other K1 bacteriophage genomes, Joy99 contains genes for virion assembly and structure, lysis proteins, host integration and excision proteins, DNA primase, RusA-like resolvase, and RtcB-like ligase.

**TABLE 1:** Characteristics of similar bacteriophages with M. smegmatis as a host^*a*^

Name	GenBank accession no.	Genome size (bp)	GC content (%)	No. of ORFs^*b*^	No. of tRNAs	Identity to Joy99 (%)
Joy99	MH536822	59,837	66.6	97	1	
BellaDonna	MH697578	59,708	66.6	97	1	99.46
Dalmuri	MH727544	59,708	66.5	97	1	99.45
Inky	MN369746	59,708	66.5	96	1	99.44
Jekyll	MF140412	59,708	66.6	97	1	99.44
CREW	KY380102	59,707	66.6	95	1	99.44

a Similarity defined as an identity of >95%.

b ORFs, open reading frames.

### Data availability.

The Joy99 genome sequence is available under GenBank accession number MH536822. The raw reads are available under SRA accession number SRX4721439.

## References

[1] World Health Organization. 2020. Global tuberculosis report 2020: executive summary. https://www.who.int/publications/i/item/9789240013131.

[2] Broxmeyer L, Sosnowska D, Miltner E, Chacón O, Wagner D, McGarvey J, Barletta RG, Bermudez LE. 2002. Killing of Mycobacterium avium and Mycobacterium tuberculosis by a mycobacteriophage delivered by a nonvirulent mycobacterium: a model for phage therapy of intracellular bacterial pathogens. J Infect Dis 186:1155–1160. doi:10.1086/343812. 12355367

[3] Allué-Guardia A, Saranathan R, Chan J, Torrelles JB. 2021. Mycobacteriophages as potential therapeutic agents against drug-resistant tuberculosis. Int J Mol Sci 22:735. doi:10.3390/ijms22020735. PMC7828454 33450990

[4] Russell DA, Hatfull GF. 2017. PhagesDB: the actinobacteriophage database. Bioinformatics 33:784–786. doi:10.1093/bioinformatics/btw711. 28365761 PMC5860397

[5] Pope WH, Bowman CA, Russell DA, Jacobs-Sera D, Asai DJ, Cresawn SG, Jacobs WR, Hendrix RW, Lawrence JG, Hatfull GF, Science Education Alliance Phage Hunters Advancing Genomics and Evolutionary Science, Phage Hunters Integrating Research and Education, Mycobacterial Genetics Course. 2015. Whole genome comparison of a large collection of mycobacteriophages reveals a continuum of phage genetic diversity. Elife 4:e06416. doi:10.7554/eLife.06416. 25919952 PMC4408529

[6] Singh AK, Reyrat J-M. 2009. Laboratory maintenance of Mycobacterium smegmatis. Curr Protoc Microbiol Chapter 10:Unit10C.1. doi:10.1002/9780471729259.mc10c01s14. 19653213

[7] Schneider CA, Rasband WS, Eliceiri KW. 2012. NIH Image to ImageJ: 25 years of image analysis. Nat Methods 9:671–675. doi:10.1038/nmeth.2089. 22930834 PMC5554542

[8] Poxleitner M, Pope W, Jacobs-Sera D, Sivanathan V, Hatfull G. 2018. Phage discovery guide. Howard Hughes Medical Institute, Chevy Chase, MD.

[9] Russell DA. 2018. Sequencing, assembling, and finishing complete bacteriophage genomes, p 109–125. *In* Clokie MRJ, Kropinski AM, Lavigne R (ed), Bacteriophages: methods and protocols, vol 3. Springer, New York, NY. 10.1007/978-1-4939-7343-9_9 29134591

[10] Gordon D, Green P. 2013. Consed: a graphical editor for next-generation sequencing. Bioinformatics 29:2936–2937. doi:10.1093/bioinformatics/btt515. 23995391 PMC3810858

[11] Altschul SF, Gish W, Miller W, Myers EW, Lipman DJ. 1990. Basic local alignment search tool. J Mol Biol 215:403–410. doi:10.1016/S0022-2836(05)80360-2. 2231712

[12] Salzberg SL, Delcher AL, Kasif S, White O. 1998. Microbial gene identification using interpolated Markov models. Nucleic Acids Res 26:544–548. doi:10.1093/nar/26.2.544. 9421513 PMC147303

[13] Borodovsky M, Mills R, Besemer J, Lomsadze A. 2003. Prokaryotic gene prediction using GeneMark and GeneMark.hmm. Curr Protoc Bioinformatics Chapter 4:Unit4.5. doi:10.1002/0471250953.bi0405s01. 18428700

[14] Söding J, Biegert A, Lupas AN. 2005. The HHpred interactive server for protein homology detection and structure prediction. Nucleic Acids Res 33:W244–W248. doi:10.1093/nar/gki408. 15980461 PMC1160169

[15] Zimmermann L, Stephens A, Nam S-Z, Rau D, Kübler J, Lozajic M, Gabler F, Söding J, Lupas AN, Alva V. 2018. A completely reimplemented MPI bioinformatics toolkit with a new HHpred server at its core. J Mol Biol 430:2237–2243. doi:10.1016/j.jmb.2017.12.007. 29258817

[16] Laslett D, Canback B. 2004. ARAGORN, a program to detect tRNA genes and tmRNA genes in nucleotide sequences. Nucleic Acids Res 32:11–16. doi:10.1093/nar/gkh152. 14704338 PMC373265

[17] Lowe TM, Chan PP. 2016. tRNAscan-SE On-line: integrating search and context for analysis of transfer RNA genes. Nucleic Acids Res 44:W54–W57. doi:10.1093/nar/gkw413. 27174935 PMC4987944

